# Supplementary material for: Diversity and structure of soil microbiota of the Jinsha earthen relic
Source: PLoS One. 2020 Jul 22;15(7):e0236165. doi: 10.1371/journal.pone.0236165 (PMC7375591; doi:10.1371/journal.pone.0236165)
Supplement: S1 Table. The primers for bacterial 16 S rRNA and fungal ITS — (DOCX) [file pone.0236165.s001.docx]

S1 Table. The primers for bacterial 16 S rRNA and fungal ITS

| primers | Primer sequence | Function |
| --- | --- | --- |
| forward primer | CCTACGGRRBGCASCAGKVRVGAAT | amplicons of the bacterial 16S rRNA V3 and V4 regions |
| reverse primer | GGACTACNVGGGTWTCTAATCC |  |
| forward primer | ACCTGCGGARGGAT | amplicons of the fungal conserved ITS1 regions |
| reverse primer | GAGATCCRTTGYTRAA |  |
| forward primer | GTGAATCATCGARTC | amplicons of the fungal conserved ITS2 regions |
| reverse primer | TCCTCCGCTTATTGAT |  |
