# Supplementary material for: Diversity and structure of soil microbiota of the Jinsha earthen relic
Source: PLoS One. 2020 Jul 22;15(7):e0236165. doi: 10.1371/journal.pone.0236165 (PMC7375591; doi:10.1371/journal.pone.0236165)
Supplement: S2 Table — (DOCX) [file pone.0236165.s002.docx]

S2 Table. Alpha diversity as measured by bacterial richness and Simpson index.

| Sample ID | Clean Tags | Effective Tags | Avg Len(bp) | OTU | ACE | Chao1 | Simpson | Shannon | Coverage |
| --- | --- | --- | --- | --- | --- | --- | --- | --- | --- |
| A12017 | 57,500 | 57,069 | 436 | 293 | 443.7792 | 392.1071 | 0.1071 | 2.9967 | 0.9987 |
| A12018 | 66,203 | 62,499 | 427 | 617 | 688.724 | 697.6522 | 0.0576 | 3.872 | 0.9982 |
| A22017 | 67,443 | 67,260 | 432 | 266 | 302.9564 | 309.0 | 0.0793 | 3.1759 | 0.9993 |
| A22018 | 69,542 | 69,289 | 419 | 383 | 458.9056 | 463.5 | 0.0282 | 4.3415 | 0.9989 |
| A32017 | 61,209 | 60,593 | 433 | 439 | 487.4372 | 481.9811 | 0.0317 | 4.1574 | 0.9988 |
| A32018 | 54,630 | 54,458 | 422 | 458 | 524.7652 | 530.85 | 0.0919 | 3.0877 | 0.9982 |
| B12017 | 55,526 | 55,293 | 436 | 544 | 680.8864 | 683.0822 | 0.0322 | 4.1251 | 0.9973 |
| B12018 | 59,055 | 59,014 | 422 | 640 | 704.8325 | 731.5385 | 0.0104 | 5.2497 | 0.9983 |
| B22017 | 44,683 | 44,566 | 434 | 522 | 637.6442 | 635.3108 | 0.0675 | 3.6341 | 0.9969 |
| B22018 | 62,525 | 60,775 | 413 | 589 | 626.5723 | 646.2195 | 0.0546 | 3.9988 | 0.9988 |
| B32017 | 41,693 | 41,289 | 434 | 683 | 808.2911 | 832.5357 | 0.0241 | 4.4411 | 0.9958 |
| B32018 | 43,433 | 36,515 | 417 | 667 | 725.0261 | 749.2692 | 0.0322 | 4.7579 | 0.9971 |
| B42017 | 43,998 | 43,577 | 435 | 629 | 745.3807 | 758.2958 | 0.0184 | 4.7254 | 0.9966 |
| B42018 | 61,473 | 61,404 | 419 | 666 | 713.2315 | 735.3333 | 0.0095 | 5.3879 | 0.9988 |
| B52017 | 44,809 | 44,695 | 434 | 438 | 560.3299 | 588.0652 | 0.0813 | 3.5342 | 0.9972 |
| B52018 | 46,364 | 46,307 | 422 | 628 | 668.6897 | 673.75 | 0.0111 | 5.3807 | 0.9984 |
| C12017 | 61,335 | 60,914 | 441 | 516 | 633.4676 | 633.6618 | 0.0974 | 3.2908 | 0.9978 |
| C12018 | 64,016 | 63,963 | 420 | 266 | 317.2623 | 319.2 | 0.1218 | 2.9212 | 0.9991 |
| C22017 | 50,208 | 50,201 | 435 | 168 | 220.0879 | 222.4737 | 0.1416 | 2.575 | 0.999 |
| C32017 | 68,404 | 63,531 | 417 | 582 | 660.2041 | 681.0 | 0.0201 | 4.6611 | 0.998 |
