# Supplementary material for: Diversity and structure of soil microbiota of the Jinsha earthen relic
Source: PLoS One. 2020 Jul 22;15(7):e0236165. doi: 10.1371/journal.pone.0236165 (PMC7375591; doi:10.1371/journal.pone.0236165)
Supplement: S3 Table — (DOCX) [file pone.0236165.s003.docx]

S3 Table. Alpha diversity as measured by fungal richness and Simpson index.

| Sample | Clean Tags | Effective Tags | AvgLen(bp) | OTU | ACE | Chao1 | Simpson | Shannon | Coverage(%) |
| --- | --- | --- | --- | --- | --- | --- | --- | --- | --- |
| A12017 | 56,774 | 56,768 | 358 | 37 | 41.5363 | 42 | 0.6376 | 0.9219 | 0.9999 |
| A12018 | 64,864 | 64,754 | 243 | 143 | 187.2451 | 170 | 0.1045 | 2.9066 | 0.9995 |
| A22017 | 47,706 | 47,683 | 357 | 23 | 26.1673 | 24.5 | 0.3599 | 1.2344 | 0.9999 |
| A22018 | 65,049 | 64,997 | 243 | 79 | 115.0894 | 106.5 | 0.0608 | 3.2391 | 0.9998 |
| A32017 | 47,281 | 47,136 | 354 | 56 | 66.1398 | 59.75 | 0.1752 | 2.1324 | 0.9998 |
| A32018 | 49,388 | 49,130 | 242 | 125 | 150.4539 | 136 | 0.0435 | 3.8057 | 0.9998 |
| B12017 | 42,863 | 42,858 | 351 | 28 | 28.9534 | 28.1429 | 0.9606 | 0.1386 | 1 |
| B12018 | 71,696 | 71,229 | 249 | 254 | 263.2244 | 264 | 0.024 | 4.6755 | 0.9999 |
| B22017 | 66,945 | 66,933 | 351 | 29 | 35.1598 | 34.25 | 0.9851 | 0.0607 | 0.9999 |
| B22018 | 35,950 | 35,922 | 239 | 333 | 340.3919 | 338 | 0.041 | 4.0038 | 0.9994 |
| B32017 | 73,705 | 73,617 | 355 | 48 | 55.314 | 53 | 0.4458 | 1.4177 | 0.9999 |
| B32018 | 65,748 | 65,480 | 257 | 77 | 88.9198 | 84.5 | 0.1757 | 2.5506 | 0.9999 |
| B42017 | 73,686 | 73,615 | 351 | 39 | 52.7224 | 53 | 0.5655 | 1.0013 | 0.9999 |
| B42018 | 66,783 | 66,012 | 253 | 60 | 96 | 74 | 0.2116 | 2.1996 | 0.9999 |
| B52017 | 78,535 | 78,326 | 351 | 34 | 35.1923 | 34.75 | 0.5392 | 1.0281 | 1 |
| B52018 | 66,673 | 65,720 | 247 | 85 | 151.0375 | 112.5 | 0.1365 | 2.7135 | 0.9998 |
| C12017 | 54,322 | 54,181 | 353 | 51 | 52.0017 | 51.25 | 0.3566 | 1.7058 | 1 |
| C12018 | 65,370 | 65,319 | 235 | 31 | 42.76 | 36.25 | 0.3989 | 1.2033 | 0.9999 |
| C22017 | 32,789 | 32,789 | 352 | 26 | 33.3029 | 31 | 0.4424 | 1.3 | 0.9998 |
| C22018 | 49,402 | 49,321 | 232 | 130 | 151.225 | 139.3333 | 0.036 | 4.0105 | 0.9998 |
| C32017 | 49,402 | 49,321 | 232 | 49 | 50.071 | 49.3333 | 0.8141 | 0.6214 | 1 |
| C32018 | 44,597 | 44,576 | 352 | 114 | 166.8664 | 126 | 0.0381 | 3.9643 | 0.9998 |
