# Supplementary material for: Diversity and structure of soil microbiota of the Jinsha earthen relic
Source: PLoS One. 2020 Jul 22;15(7):e0236165. doi: 10.1371/journal.pone.0236165 (PMC7375591; doi:10.1371/journal.pone.0236165)
Supplement: S4 Table — (DOCX) [file pone.0236165.s004.docx]

Table S4 The characteristics of environmental factors

|  | SWC(%) | ST (°C) | SC(μs/cm) | SSC(mg/L) | IAT (°C) | IAH (%) |
| --- | --- | --- | --- | --- | --- | --- |
| site A in 2017 | 17.51 | 20 | 896 | 519.7 | 18.2 | 70 |
| site A in 2018 | 15.12 | 21.6 | 896 | 519.7 | 20.5 | 57 |
| site B in 2017 | 4.59 | 19.6 | 308 | 159.4 | 18.2 | 70 |
| site B in 2018 | 4.81 | 20.2 | 308 | 159.4 | 20.5 | 57 |
| site C in 2017 | 5.13 | 19.5 | 2 | 0.7 | 18.2 | 70 |
| site C in 2018 | 4.65 | 20.9 | 2 | 0.7 | 20.5 | 57 |

SWC: soil water content;T: Soil Temperature; SC: soil conductivity; SSC: soil salt contents ; IAT : indoor air temperature; IAH :Indoor air humidity.
