# Supplementary material for: Diversity and structure of soil microbiota of the Jinsha earthen relic
Source: PLoS One. 2020 Jul 22;15(7):e0236165. doi: 10.1371/journal.pone.0236165 (PMC7375591; doi:10.1371/journal.pone.0236165)
Supplement: S2 Fig — Venn diagram showing the unique and shared OTUs (97%) for the fungal communities in 2017 (A) and in 2018 (B). (DOCX) [file pone.0236165.s006.docx]

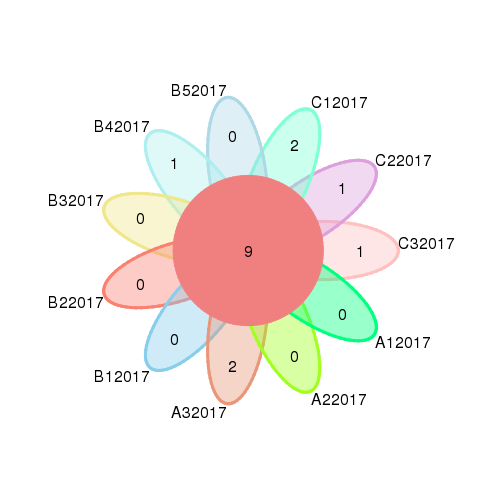
A
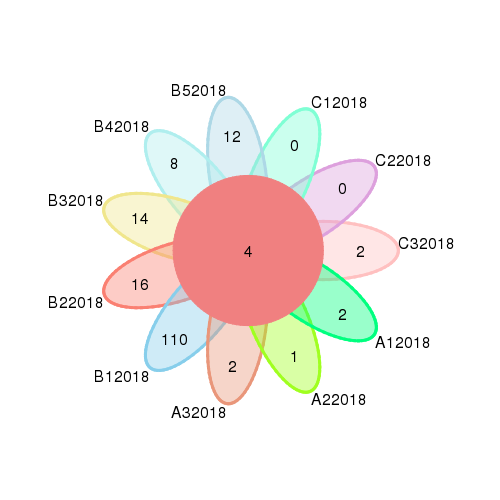
B

S2 Fig. Shared OTU analysis of the different samples. Venn diagram showing the unique and shared OTUs (97%) for the fungi in 2017 (A) and 2018 (B) communities among the 22 samples.
