# Supplementary figures and images for: Diversity and structure of soil microbiota of the Jinsha earthen relic
Source: PLoS One. 2020 Jul 22;15(7):e0236165. doi: 10.1371/journal.pone.0236165 (PMC7375591; doi:10.1371/journal.pone.0236165)

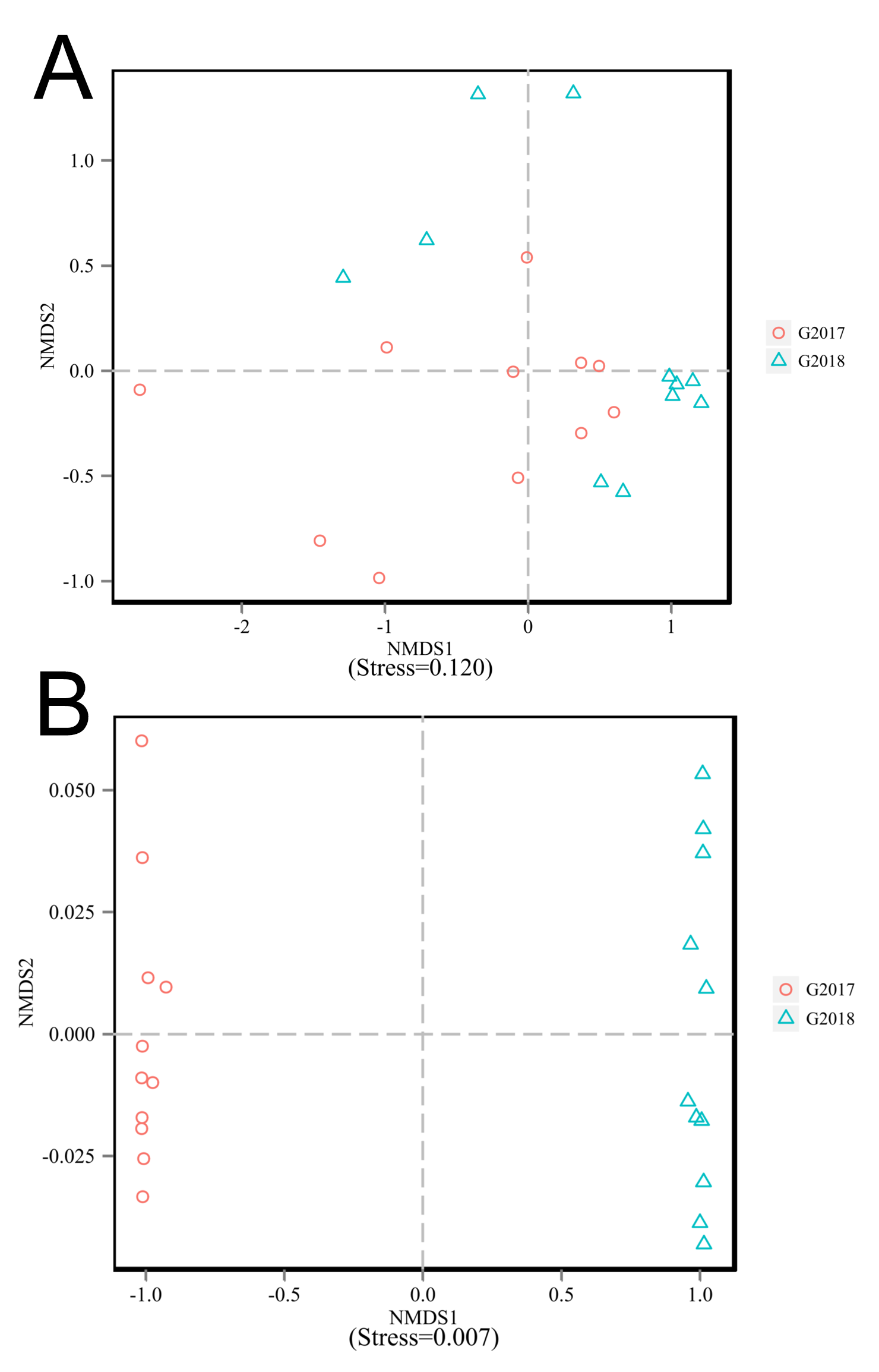


S3 Fig. NMDS analysis of bacterial and fungal communities in 2017 and 2018

Supplement: S3 Fig — NMDS analysis of bacterial and fungal communities in 2017 (A) and in 2018 (B). (DOCX) [file pone.0236165.s007.docx]
