# Supplementary material for: Diversity and structure of soil microbiota of the Jinsha earthen relic
Source: PLoS One. 2020 Jul 22;15(7):e0236165. doi: 10.1371/journal.pone.0236165 (PMC7375591; doi:10.1371/journal.pone.0236165)
Supplement: S4 Fig — Principal component analyses of the bacterial (A) communities in the 22 samples in 2017 and 2018. Weighted UniFrac UPGMA tree based on the bacteria V3+V4 rRNA gene sequences. (DOCX) [file pone.0236165.s008.docx]

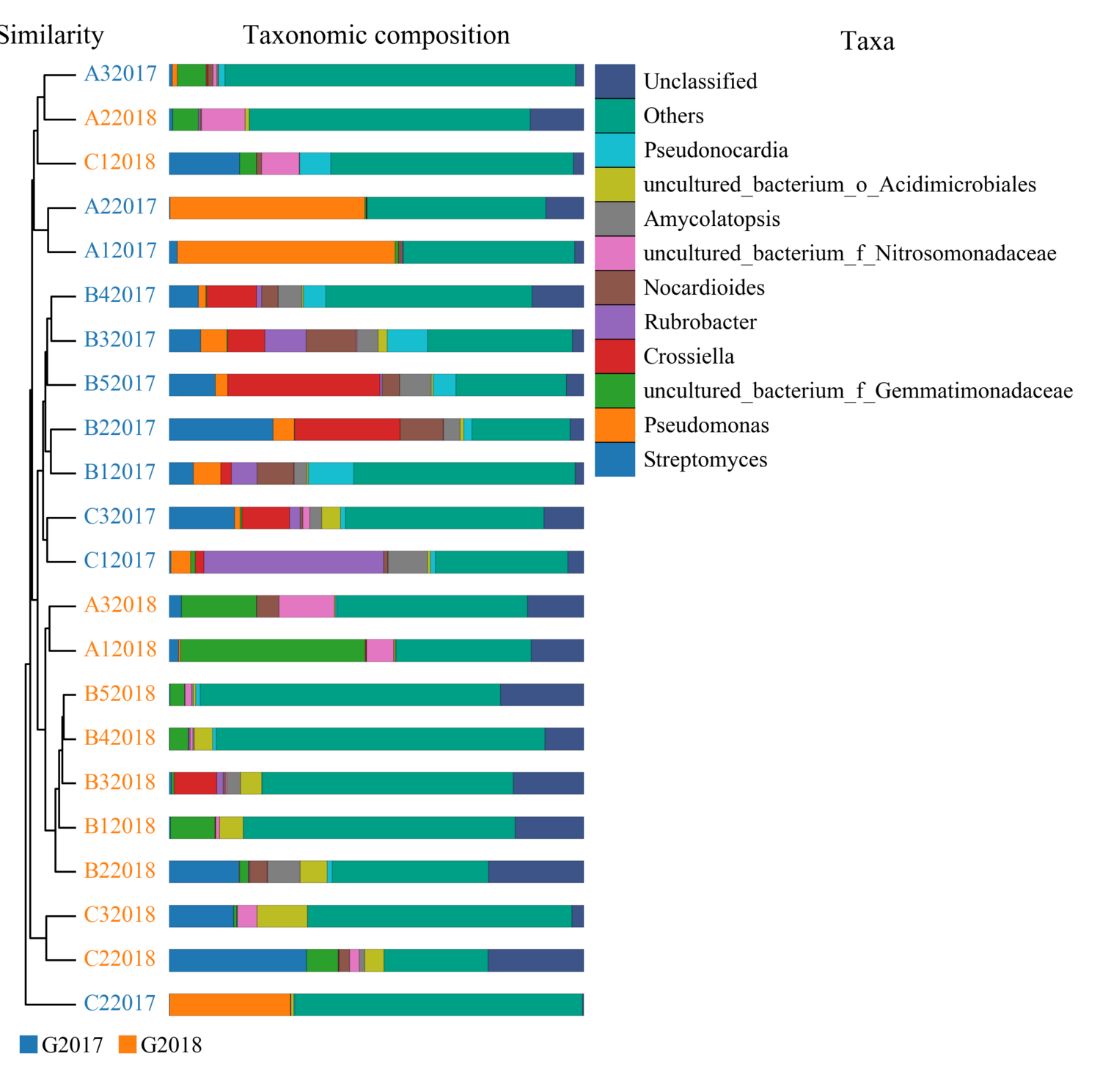


S4 Fig. Principal component analyses of the bacterial (A) communities in the 22 samples in 2017 and 2018.Weighted UniFrac UPGMA tree based on bacteria V3+V4 gene sequences.
