# Supplementary material for: Diversity and structure of soil microbiota of the Jinsha earthen relic
Source: PLoS One. 2020 Jul 22;15(7):e0236165. doi: 10.1371/journal.pone.0236165 (PMC7375591; doi:10.1371/journal.pone.0236165)
Supplement: S5 Fig — Weighted UniFrac UPGMA tree based on fungal ITS gene sequences. (DOCX) [file pone.0236165.s009.docx]

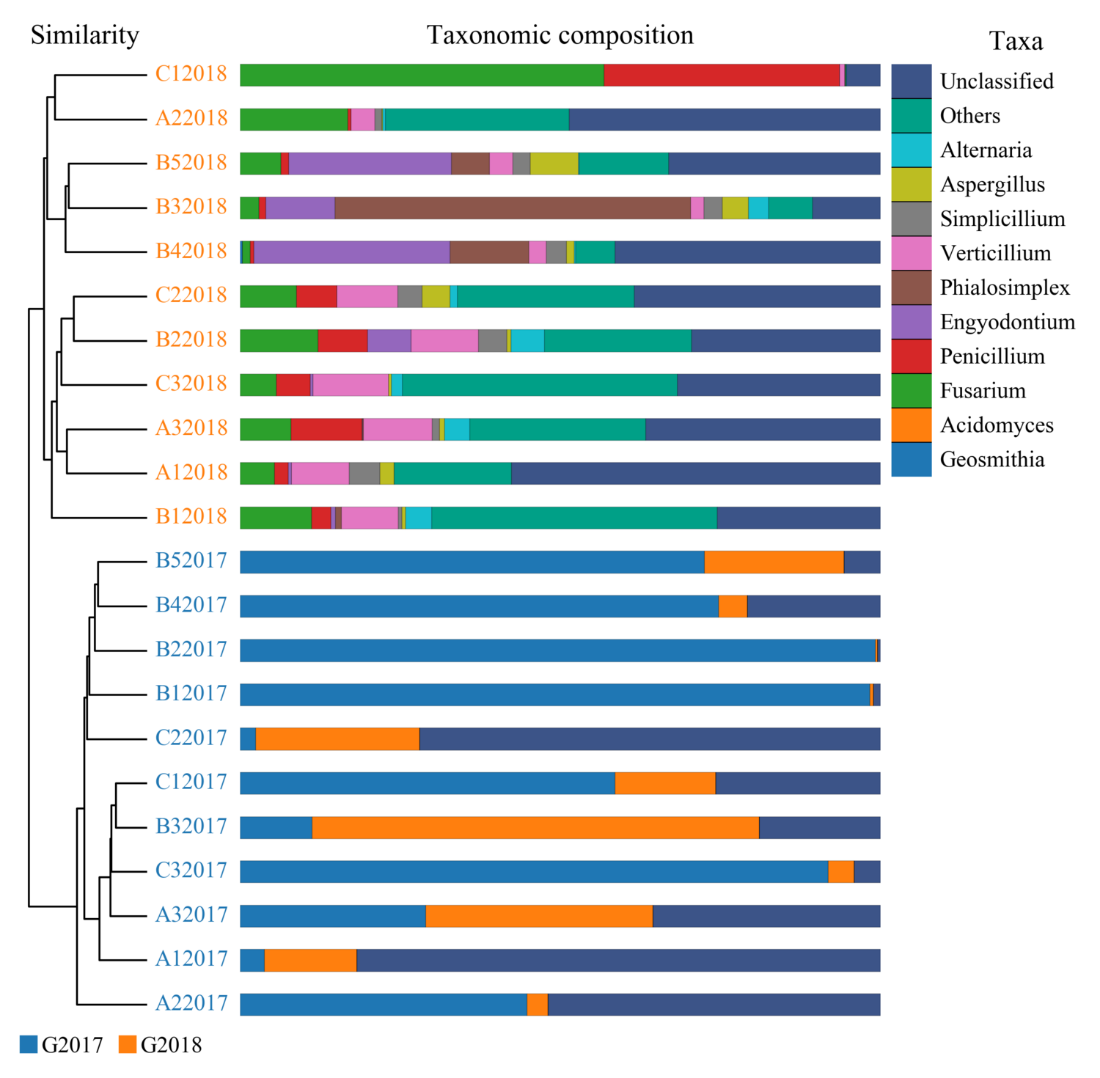


S5 Fig. Principal component analyses of the fungal communities in the 22 samples in 2017 and 2018.Weighted UniFrac UPGMA tree based on fungal ITS gene sequences.
