# Supplementary material for: Diversity and structure of soil microbiota of the Jinsha earthen relic
Source: PLoS One. 2020 Jul 22;15(7):e0236165. doi: 10.1371/journal.pone.0236165 (PMC7375591; doi:10.1371/journal.pone.0236165)
Supplement: Attachment — Submitted filename: PONE-D-20-03599-Reviewer Attachment.doc [file pone.0236165.s010.doc]

The manuscript investigated diversity and structure of soil microbiota of the Jinsha earthen relic. Authors analyzed 22 soil samples from the Jinsha earthen relic in China during 2017 and 2018. In addition, predominant microorganisms were isolated and identified from samples by molecular biological analysis technology. This study is very interesting, however, some questions are still required to be discussed here.

1. The author only collected soil of different ages at site B, and whether site A and C should be consistent, and what age groups.
2. The site B and C were perennially dry. The conditions such as climate, temperature, humidity, latitude and longitude at the time of sampling, and the method of storage and transportation of the sample will affect the results. Has the author considered the above factors?
3. Line 181~183: It is recommended to analysis of fungal diversity in different years in the same area.
4. Line 335~336:＂remained the most populous strain in samples from 2018, although other strains were also detected in these soil samples from 2018, showing that the diversity of the fungal species increased over time.＂Whether there is regularity in two years?
5. Line 114: Please provide the information of software and its version used for PCA.
6. The author only talked about the sequencing results of bacteria in different regions, but did not combine it with damage degree of soil, nor did not explore whether different strains caused different damage, which is extremely important.
7. Whether there are chemical substances in soil samples participating in the metabolism and decomposition of main microorganisms? This problem needs to be explained in the introduction of the article.
