# Supplementary material for: Diversity and structure of soil microbiota of the Jinsha earthen relic
Source: PLoS One. 2020 Jul 22;15(7):e0236165. doi: 10.1371/journal.pone.0236165 (PMC7375591; doi:10.1371/journal.pone.0236165)
Supplement: Attachment — Submitted filename: PONE-D-20-03599_reviewer.docx [file pone.0236165.s011.docx]

The soil microbiota and mycobiota from Jinsha earthen relic were analyzed using NGS technique and bioinformatic analysis. Major goal and concept of this study are very interesting and have an importance to suggest guideline to prevent microbial corrosion. But, some questions are still required to be discussed here.

1. Please check again misprints in whole manuscript and improve a figure resolution
2. The major goal of this study analyze soil microbiota and mycobiota of earthen relic and provide a data which can use to develop the guide for prevention and control of microbial corrosion. To support a major goal of this study, it is necessary that correlation analysis using soil microbiome data, states of unearthed artifact, and metadata of sampling site such as temperature, humidity, and climate is need.
3. 122~125: In OTU clustering and taxonomic assignment step, SILVA and Greengene database were used respectively. But, it is not well known that how much different taxonomic assignment result when two databases were used at once. So, please mention the difference of taxonomic assignment via SILVA and Greengene database to confirm an approach for OTU clustering and taxonomic assignment step.
4. 165~185: To demonstrate a major concept of this study well, it is easier to understand that calculate a diversity data of soil microbiota and mycobiota based on the year or sampling site than individual samples.
5. 234~241: It is good approach that analyze beta diversity by sample group. But, there is no mention the method to calculate a distance matrix in beta diversity. Furthermore, it is hard to say whether two groups are distinguished or not by PCA analysis. So, NMDS or PERMANOVA analysis should be need to support this result additionally.
6. 255~263: The strategy that analyze the factor to cause environmental change using microbial function from soil microbiota is good. But the data which related to this is generated using level 1 result of PICRUSt. In Discussion part, microbial function which similar with level 2 or 3 result of PICRUSt and its bacterial phylum were mention together. To support this, additional PICRUSt data via level 3 result may need.
